# Hexa­kis­(dimethyl sulfoxide-κ*O*)calcium μ_6_-oxido-dodeca­kis-μ_2_-oxido-hexa­oxido­hexa­tungstate(VI)

**DOI:** 10.1107/S1600536812018338

**Published:** 2012-04-28

**Authors:** Jinfang Zhang

**Affiliations:** a Molecular Materials Research Center, Scientific Research Academy, School of Chemistry and Chemical Engineering, Jiangsu University, Zhenjiang 212013, People’s Republic of China

## Abstract

In the title compound, [Ca(C_2_H_6_OS)_6_][W_6_O_19_], the cation and anion both have a crystallographically imposed centre of symmetry. The Ca^II^ atom in the cation is coordinated by six O atoms from six dimethyl sulfoxide ligands in a distorted octa­hedral geometry. The [W_6_O_19_]^2−^ isopolyanion possesses the well-known Lindqvist structure in which each W^VI^ atom is coordinated by four μ_2_-O, one terminal O and one μ_6_-O atom.

## Related literature

For the *in situ* synthetic method, see: Xu *et al.* (2010 ▶); Ni *et al.* (2009 ▶).
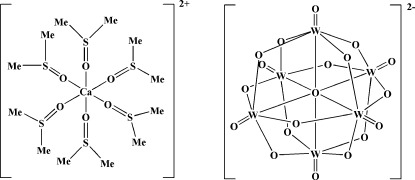


## Experimental

### 

#### Crystal data

[Ca(C_2_H_6_OS)_6_][W_6_O_19_]
*M* *_r_* = 1915.95
Triclinic, 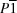

*a* = 8.1871 (16) Å
*b* = 11.352 (2) Å
*c* = 11.378 (2) Å
α = 84.53 (3)°
β = 73.15 (3)°
γ = 74.02 (3)°
*V* = 972.8 (3) Å^3^
*Z* = 1
Mo *K*α radiation
μ = 18.20 mm^−1^
*T* = 293 K
0.21 × 0.15 × 0.13 mm

#### Data collection

Rigaku Saturn724+ diffractometer
Absorption correction: multi-scan (*CrystalClear*; Rigaku, 2008 ▶) *T*_min_ = 0.047, *T*_max_ = 0.094
8635 measured reflections
3507 independent reflections
3072 reflections with *I* > 2σ(*I*)
*R*_int_ = 0.034

#### Refinement

*R*[*F* ^2^ > 2σ(*F* ^2^)] = 0.023
*wR*(*F* ^2^) = 0.047
*S* = 0.98
3507 reflections
229 parameters
H-atom parameters constrained
Δρ_max_ = 1.05 e Å^−3^
Δρ_min_ = −1.19 e Å^−3^

### 

Data collection: *CrystalClear* (Rigaku, 2008 ▶); cell refinement: *CrystalClear*; data reduction: *CrystalClear*; program(s) used to solve structure: *SHELXTL* (Sheldrick, 2008 ▶); program(s) used to refine structure: *SHELXTL*; molecular graphics: *SHELXTL*; software used to prepare material for publication: *SHELXTL*.

## Supplementary Material

Crystal structure: contains datablock(s) I, global. DOI: 10.1107/S1600536812018338/rz2743sup1.cif

Structure factors: contains datablock(s) I. DOI: 10.1107/S1600536812018338/rz2743Isup2.hkl

Additional supplementary materials:  crystallographic information; 3D view; checkCIF report

## Figures and Tables

**Fig. 1.**
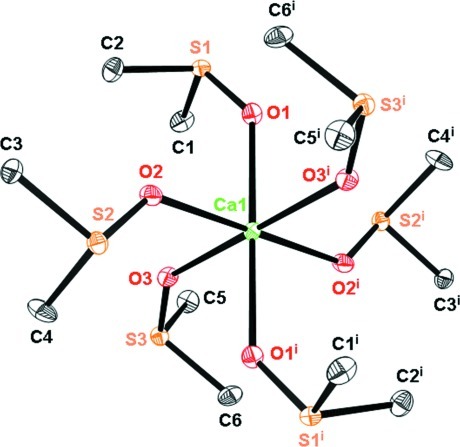
The molecular structure of the cation in the title compound, with 30% probability displacement ellipsoids. H atoms have been omitted. Symmetry code: (i) -x, 1 - y, 1 - z.

**Fig. 2.**
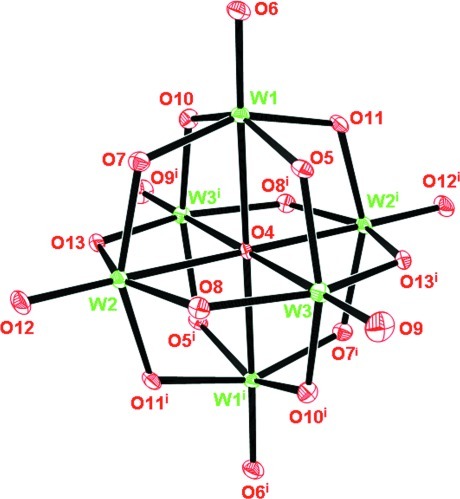
The molecular structure of the anion in the title compound, with 30% probability displacement ellipsoids. Symmetry code: (i) 1 - x, -y, -z.

## supplementary crystallographic information

### Comment

The *in situ* synthetic method has been widely applied to constructing new compounds (Xu *et al.*, 2010, Ni *et al.*, 2009). In the present paper, the *in situ* reaction between tetrathiotungstate and calcium nitrate in DMSO to form the title compound is reported. The (W_6_O_19_)^2-^ isopolyanion was achieved by the reaction between (WS_4_)^2-^ and H_2_O in DMSO solution.

In the cation of the title compound (Fig. 1), the Ca^2+^ ion is bonded by six O atoms from six DMSO ligands in a distorted octahedral coordination geometry. The (W_6_O_19_)^2-^ (Fig. 2) shows the usual cage-shaped Lindqvist structure, where each W atom is coordinated by four *µ*_2_-O, one terminal O and one *µ*_6_-O atoms. The W—O bond lengths involving the µ_6_-O atoms [2.3255 (8)–2.3292 (6) Å] are obviously longer than those observed for the µ_2_-O [1.905 (4)–1.934 (4) Å] and terminal [1.696 (4)–1.703 (4) Å] oxygen atoms.

### Experimental

Calcium nitrate (1 mmol) was added to a solution of [NH_4_]_2_WS_4_ (1 mmol in 5 ml DMSO) with thorough stir for 30 minutes. After filtration, the orange filtrate was carefully laid on the surface with 8 ml *i*-PrOH. Colourless block crystals were obtained after about one month.

### Refinement

H atoms were positioned geometrically and refined using a riding model, with C—H = 0.96 Å and with *U*_iso_(H) = 1.5*U*_eq_(C).

### Crystal data

**Table:**

[Ca(C_2_H_6_OS)_6_][W_6_O_19_]	*Z* = 1
*M**_r_* = 1915.95	*F*(000) = 868
Triclinic, *P*1	*D*_x_ = 3.270 Mg m^−^^3^
Hall symbol: -P 1	Mo *K*α radiation, λ = 0.71073 Å
*a* = 8.1871 (16) Å	Cell parameters from 4353 reflections
*b* = 11.352 (2) Å	θ = 3.7–29.0°
*c* = 11.378 (2) Å	µ = 18.20 mm^−^^1^
α = 84.53 (3)°	*T* = 293 K
β = 73.15 (3)°	Block, colourless
γ = 74.02 (3)°	0.21 × 0.15 × 0.13 mm
*V* = 972.8 (3) Å^3^	

### Data collection

**Table:**

Rigaku Saturn724+ diffractometer	3507 independent reflections
Radiation source: fine-focus sealed tube	3072 reflections with *I* > 2σ(*I*)
Graphite monochromator	*R*_int_ = 0.034
ω scans	θ_max_ = 25.4°, θ_min_ = 3.7°
Absorption correction: multi-scan (*CrystalClear*; Rigaku, 2008)	*h* = −9→9
*T*_min_ = 0.047, *T*_max_ = 0.094	*k* = −12→13
8635 measured reflections	*l* = −13→12

### Refinement

**Table:**

Refinement on *F*^2^	Primary atom site location: structure-invariant direct methods
Least-squares matrix: full	Secondary atom site location: difference Fourier map
*R*[*F*^2^ > 2σ(*F*^2^)] = 0.023	Hydrogen site location: inferred from neighbouring sites
*wR*(*F*^2^) = 0.047	H-atom parameters constrained
*S* = 0.98	*w* = 1/[σ^2^(*F*_o_^2^) + (0.0163*P*)^2^] where *P* = (*F*_o_^2^ + 2*F*_c_^2^)/3
3507 reflections	(Δ/σ)_max_ = 0.001
229 parameters	Δρ_max_ = 1.05 e Å^−^^3^
0 restraints	Δρ_min_ = −1.19 e Å^−^^3^

### Special details

**Table:**

Geometry. All e.s.d.'s (except the e.s.d. in the dihedral angle between two l.s. planes) are estimated using the full covariance matrix. The cell e.s.d.'s are taken into account individually in the estimation of e.s.d.'s in distances, angles and torsion angles; correlations between e.s.d.'s in cell parameters are only used when they are defined by crystal symmetry. An approximate (isotropic) treatment of cell e.s.d.'s is used for estimating e.s.d.'s involving l.s. planes.
Refinement. Refinement of *F*^2^ against ALL reflections. The weighted *R*-factor *wR* and goodness of fit *S* are based on *F*^2^, conventional *R*-factors *R* are based on *F*, with *F* set to zero for negative *F*^2^. The threshold expression of *F*^2^ > σ(*F*^2^) is used only for calculating *R*-factors(gt) *etc*. and is not relevant to the choice of reflections for refinement. *R*-factors based on *F*^2^ are statistically about twice as large as those based on *F*, and *R*- factors based on ALL data will be even larger.

### Fractional atomic coordinates and isotropic or equivalent isotropic displacement parameters (Å^2^)

**Table:**

	*x*	*y*	*z*	*U*_iso_*/*U*_eq_	
Ca1	0.0000	0.5000	0.5000	0.0163 (4)	
S1	−0.2595 (2)	0.38529 (14)	0.35230 (14)	0.0203 (3)	
S2	0.2007 (2)	0.23054 (14)	0.63729 (14)	0.0206 (3)	
S3	0.3557 (2)	0.47593 (14)	0.21704 (13)	0.0205 (4)	
O1	−0.2315 (5)	0.4680 (4)	0.4393 (3)	0.0232 (10)	
O2	0.0581 (5)	0.3034 (4)	0.5797 (4)	0.0250 (10)	
O3	0.2119 (5)	0.4354 (4)	0.3150 (3)	0.0224 (10)	
C1	−0.1301 (9)	0.4158 (6)	0.2049 (5)	0.0302 (16)	
H1A	−0.1824	0.4963	0.1777	0.045*	
H1B	−0.1247	0.3562	0.1485	0.045*	
H1C	−0.0129	0.4115	0.2085	0.045*	
C2	−0.1315 (9)	0.2352 (6)	0.3743 (6)	0.0293 (16)	
H2A	−0.1852	0.2032	0.4529	0.044*	
H2B	−0.0142	0.2379	0.3713	0.044*	
H2C	−0.1259	0.1831	0.3107	0.044*	
C3	0.1723 (8)	0.0790 (5)	0.6536 (6)	0.0251 (15)	
H3A	0.0671	0.0769	0.7180	0.038*	
H3B	0.2726	0.0233	0.6737	0.038*	
H3C	0.1619	0.0553	0.5779	0.038*	
C4	0.4000 (8)	0.2041 (6)	0.5156 (6)	0.0321 (17)	
H4A	0.4348	0.2792	0.4944	0.048*	
H4B	0.3812	0.1750	0.4453	0.048*	
H4C	0.4913	0.1439	0.5415	0.048*	
C5	0.2577 (9)	0.5451 (6)	0.0969 (5)	0.0299 (16)	
H5A	0.2362	0.4830	0.0561	0.045*	
H5B	0.3366	0.5856	0.0388	0.045*	
H5C	0.1480	0.6039	0.1314	0.045*	
C6	0.3767 (9)	0.6117 (6)	0.2698 (6)	0.0290 (16)	
H6A	0.4292	0.5919	0.3368	0.044*	
H6B	0.2619	0.6676	0.2970	0.044*	
H6C	0.4502	0.6492	0.2041	0.044*	
W1	0.29354 (3)	0.17964 (2)	−0.02810 (2)	0.01538 (7)	
W2	0.34391 (3)	−0.00577 (2)	0.20593 (2)	0.01614 (8)	
W3	0.66800 (3)	0.11373 (2)	0.04884 (2)	0.01671 (8)	
O4	0.5000	0.0000	0.0000	0.0149 (12)	
O5	0.4702 (5)	0.2359 (3)	0.0143 (3)	0.0159 (9)	
O6	0.1471 (5)	0.3129 (4)	−0.0504 (4)	0.0237 (10)	
O7	0.2105 (5)	0.1407 (4)	0.1419 (3)	0.0182 (9)	
O8	0.5081 (5)	0.0881 (4)	0.2053 (3)	0.0161 (9)	
O9	0.7883 (6)	0.1969 (4)	0.0863 (4)	0.0286 (11)	
O10	0.2012 (5)	0.0534 (4)	−0.0611 (3)	0.0181 (9)	
O11	0.4597 (5)	0.1488 (3)	−0.1889 (3)	0.0178 (9)	
O12	0.2325 (5)	−0.0106 (4)	0.3573 (3)	0.0238 (10)	
O13	0.2413 (5)	−0.0963 (4)	0.1266 (3)	0.0183 (9)	

### Atomic displacement parameters (Å^2^)

**Table:**

	*U*^11^	*U*^22^	*U*^33^	*U*^12^	*U*^13^	*U*^23^
Ca1	0.0190 (9)	0.0153 (10)	0.0149 (8)	−0.0052 (8)	−0.0050 (7)	0.0023 (7)
S1	0.0193 (8)	0.0218 (9)	0.0217 (8)	−0.0076 (7)	−0.0067 (7)	0.0002 (7)
S2	0.0207 (8)	0.0197 (9)	0.0207 (8)	−0.0029 (7)	−0.0075 (7)	0.0012 (7)
S3	0.0203 (8)	0.0207 (9)	0.0183 (8)	−0.0051 (7)	−0.0027 (7)	0.0013 (7)
O1	0.025 (2)	0.025 (3)	0.020 (2)	−0.004 (2)	−0.0088 (19)	−0.0027 (19)
O2	0.020 (2)	0.020 (2)	0.035 (2)	−0.005 (2)	−0.011 (2)	0.009 (2)
O3	0.026 (2)	0.023 (3)	0.017 (2)	−0.010 (2)	0.0001 (19)	0.0023 (18)
C1	0.041 (4)	0.035 (4)	0.019 (3)	−0.018 (3)	−0.007 (3)	0.000 (3)
C2	0.037 (4)	0.022 (4)	0.030 (4)	−0.008 (3)	−0.012 (3)	0.003 (3)
C3	0.027 (4)	0.018 (4)	0.031 (4)	−0.006 (3)	−0.009 (3)	0.003 (3)
C4	0.016 (4)	0.044 (5)	0.032 (4)	−0.008 (3)	−0.003 (3)	0.009 (3)
C5	0.036 (4)	0.031 (4)	0.023 (4)	−0.010 (3)	−0.008 (3)	0.006 (3)
C6	0.033 (4)	0.032 (4)	0.025 (4)	−0.020 (3)	−0.003 (3)	0.003 (3)
W1	0.01630 (14)	0.01265 (14)	0.01563 (13)	−0.00101 (10)	−0.00495 (10)	0.00088 (10)
W2	0.01678 (14)	0.01716 (15)	0.01205 (13)	−0.00261 (11)	−0.00237 (10)	0.00101 (10)
W3	0.01879 (14)	0.01511 (15)	0.01880 (14)	−0.00643 (11)	−0.00742 (10)	0.00045 (10)
O4	0.013 (3)	0.015 (3)	0.014 (3)	−0.005 (2)	−0.001 (2)	0.006 (2)
O5	0.022 (2)	0.011 (2)	0.014 (2)	−0.0026 (18)	−0.0071 (18)	0.0002 (17)
O6	0.025 (2)	0.019 (2)	0.023 (2)	−0.001 (2)	−0.0060 (19)	0.0046 (19)
O7	0.019 (2)	0.017 (2)	0.017 (2)	−0.0013 (18)	−0.0047 (18)	−0.0019 (18)
O8	0.021 (2)	0.017 (2)	0.0113 (19)	−0.0040 (18)	−0.0053 (17)	−0.0049 (17)
O9	0.031 (3)	0.027 (3)	0.037 (3)	−0.015 (2)	−0.015 (2)	−0.004 (2)
O10	0.019 (2)	0.018 (2)	0.020 (2)	−0.0067 (18)	−0.0061 (18)	−0.0017 (18)
O11	0.022 (2)	0.015 (2)	0.015 (2)	−0.0046 (19)	−0.0057 (18)	0.0052 (17)
O12	0.028 (3)	0.027 (3)	0.011 (2)	−0.004 (2)	−0.0014 (19)	0.0024 (18)
O13	0.016 (2)	0.021 (2)	0.019 (2)	−0.0080 (19)	−0.0049 (18)	0.0077 (18)

### Geometric parameters (Å, º)

**Table:**

S1—O1	1.530 (4)	C6—H6C	0.9600
S1—C1	1.765 (6)	W1—O6	1.701 (4)
S1—C2	1.776 (6)	W1—O10	1.905 (4)
S2—O2	1.512 (4)	W1—O7	1.907 (4)
S2—C4	1.780 (6)	W1—O5	1.924 (4)
S2—C3	1.784 (6)	W1—O11	1.934 (4)
S3—O3	1.509 (4)	W1—O4	2.3258 (9)
S3—C6	1.775 (6)	W2—O12	1.703 (4)
S3—C5	1.792 (6)	W2—O13	1.916 (4)
C1—H1A	0.9600	W2—O11^i^	1.924 (4)
C1—H1B	0.9600	W2—O7	1.929 (4)
C1—H1C	0.9600	W2—O8	1.930 (4)
C2—H2A	0.9600	W2—O4	2.3255 (8)
C2—H2B	0.9600	W3—O9	1.696 (4)
C2—H2C	0.9600	W3—O10^i^	1.919 (4)
C3—H3A	0.9600	W3—O13^i^	1.926 (4)
C3—H3B	0.9600	W3—O5	1.931 (4)
C3—H3C	0.9600	W3—O8	1.931 (4)
C4—H4A	0.9600	W3—O4	2.3292 (6)
C4—H4B	0.9600	O4—W2^i^	2.3255 (8)
C4—H4C	0.9600	O4—W1^i^	2.3258 (9)
C5—H5A	0.9600	O4—W3^i^	2.3292 (6)
C5—H5B	0.9600	O10—W3^i^	1.919 (4)
C5—H5C	0.9600	O11—W2^i^	1.924 (4)
C6—H6A	0.9600	O13—W3^i^	1.926 (4)
C6—H6B	0.9600		
			
O1—S1—C1	105.5 (3)	O10—W1—O4	76.19 (12)
O1—S1—C2	106.5 (3)	O7—W1—O4	76.43 (12)
C1—S1—C2	98.3 (3)	O5—W1—O4	76.06 (11)
O2—S2—C4	105.3 (3)	O11—W1—O4	76.15 (12)
O2—S2—C3	104.4 (3)	O12—W2—O13	104.30 (18)
C4—S2—C3	98.5 (3)	O12—W2—O11^i^	103.04 (18)
O3—S3—C6	106.8 (3)	O13—W2—O11^i^	86.91 (16)
O3—S3—C5	106.0 (3)	O12—W2—O7	104.58 (18)
C6—S3—C5	97.5 (3)	O13—W2—O7	87.18 (16)
S1—C1—H1A	109.5	O11^i^—W2—O7	152.37 (16)
S1—C1—H1B	109.5	O12—W2—O8	102.96 (18)
H1A—C1—H1B	109.5	O13—W2—O8	152.74 (15)
S1—C1—H1C	109.5	O11^i^—W2—O8	86.82 (16)
H1A—C1—H1C	109.5	O7—W2—O8	86.18 (17)
H1B—C1—H1C	109.5	O12—W2—O4	179.15 (14)
S1—C2—H2A	109.5	O13—W2—O4	76.27 (11)
S1—C2—H2B	109.5	O11^i^—W2—O4	76.33 (11)
H2A—C2—H2B	109.5	O7—W2—O4	76.04 (11)
S1—C2—H2C	109.5	O8—W2—O4	76.47 (11)
H2A—C2—H2C	109.5	O9—W3—O10^i^	104.34 (19)
H2B—C2—H2C	109.5	O9—W3—O13^i^	104.75 (19)
S2—C3—H3A	109.5	O10^i^—W3—O13^i^	86.68 (17)
S2—C3—H3B	109.5	O9—W3—O5	103.97 (19)
H3A—C3—H3B	109.5	O10^i^—W3—O5	151.69 (16)
S2—C3—H3C	109.5	O13^i^—W3—O5	85.75 (16)
H3A—C3—H3C	109.5	O9—W3—O8	102.89 (18)
H3B—C3—H3C	109.5	O10^i^—W3—O8	87.37 (16)
S2—C4—H4A	109.5	O13^i^—W3—O8	152.35 (16)
S2—C4—H4B	109.5	O5—W3—O8	86.80 (16)
H4A—C4—H4B	109.5	O9—W3—O4	179.23 (15)
S2—C4—H4C	109.5	O10^i^—W3—O4	75.84 (11)
H4A—C4—H4C	109.5	O13^i^—W3—O4	76.00 (11)
H4B—C4—H4C	109.5	O5—W3—O4	75.85 (11)
S3—C5—H5A	109.5	O8—W3—O4	76.36 (11)
S3—C5—H5B	109.5	W2—O4—W2^i^	180.000 (15)
H5A—C5—H5B	109.5	W2—O4—W1^i^	90.19 (4)
S3—C5—H5C	109.5	W2^i^—O4—W1^i^	89.81 (4)
H5A—C5—H5C	109.5	W2—O4—W1	89.81 (4)
H5B—C5—H5C	109.5	W2^i^—O4—W1	90.19 (4)
S3—C6—H6A	109.5	W1^i^—O4—W1	180.000 (18)
S3—C6—H6B	109.5	W2—O4—W3	90.07 (3)
H6A—C6—H6B	109.5	W2^i^—O4—W3	89.93 (3)
S3—C6—H6C	109.5	W1^i^—O4—W3	89.690 (19)
H6A—C6—H6C	109.5	W1—O4—W3	90.310 (19)
H6B—C6—H6C	109.5	W2—O4—W3^i^	89.93 (3)
O6—W1—O10	105.04 (19)	W2^i^—O4—W3^i^	90.07 (3)
O6—W1—O7	104.53 (18)	W1^i^—O4—W3^i^	90.310 (19)
O10—W1—O7	87.11 (16)	W1—O4—W3^i^	89.690 (19)
O6—W1—O5	102.69 (18)	W3—O4—W3^i^	180.000 (12)
O10—W1—O5	152.24 (17)	W1—O5—W3	117.76 (19)
O7—W1—O5	87.11 (16)	W1—O7—W2	117.72 (19)
O6—W1—O11	102.88 (18)	W2—O8—W3	117.10 (18)
O10—W1—O11	86.69 (16)	W1—O10—W3^i^	118.3 (2)
O7—W1—O11	152.57 (17)	W2^i^—O11—W1	117.33 (19)
O5—W1—O11	86.06 (16)	W2—O13—W3^i^	117.79 (18)
O6—W1—O4	178.43 (14)		
			
O13—W2—O4—W1^i^	−89.97 (12)	O8—W3—O4—W1	89.34 (13)
O11^i^—W2—O4—W1^i^	0.16 (12)	O6—W1—O5—W3	179.93 (19)
O7—W2—O4—W1^i^	179.54 (12)	O10—W1—O5—W3	−2.5 (4)
O8—W2—O4—W1^i^	90.15 (12)	O7—W1—O5—W3	75.7 (2)
O13—W2—O4—W1	90.03 (12)	O11—W1—O5—W3	−77.7 (2)
O11^i^—W2—O4—W1	−179.84 (12)	O4—W1—O5—W3	−1.05 (15)
O7—W2—O4—W1	−0.46 (12)	O9—W3—O5—W1	−178.2 (2)
O8—W2—O4—W1	−89.85 (12)	O10^i^—W3—O5—W1	2.7 (4)
O13—W2—O4—W3	−179.66 (12)	O13^i^—W3—O5—W1	77.7 (2)
O11^i^—W2—O4—W3	−89.53 (12)	O8—W3—O5—W1	−75.7 (2)
O7—W2—O4—W3	89.85 (12)	O4—W3—O5—W1	1.05 (15)
O8—W2—O4—W3	0.46 (12)	O6—W1—O7—W2	−179.4 (2)
O13—W2—O4—W3^i^	0.34 (12)	O10—W1—O7—W2	75.8 (2)
O11^i^—W2—O4—W3^i^	90.47 (12)	O5—W1—O7—W2	−77.0 (2)
O7—W2—O4—W3^i^	−90.15 (12)	O11—W1—O7—W2	−1.3 (5)
O8—W2—O4—W3^i^	−179.54 (12)	O4—W1—O7—W2	−0.64 (16)
O10—W1—O4—W2	−89.87 (12)	O12—W2—O7—W1	−180.0 (2)
O7—W1—O4—W2	0.47 (12)	O13—W2—O7—W1	−75.9 (2)
O5—W1—O4—W2	90.83 (11)	O11^i^—W2—O7—W1	1.9 (5)
O11—W1—O4—W2	−179.84 (12)	O8—W2—O7—W1	77.6 (2)
O10—W1—O4—W2^i^	90.13 (12)	O4—W2—O7—W1	0.64 (16)
O7—W1—O4—W2^i^	−179.53 (12)	O12—W2—O8—W3	178.7 (2)
O5—W1—O4—W2^i^	−89.17 (11)	O13—W2—O8—W3	−0.9 (5)
O11—W1—O4—W2^i^	0.16 (12)	O11^i^—W2—O8—W3	76.1 (2)
O10—W1—O4—W3	−179.94 (11)	O7—W2—O8—W3	−77.2 (2)
O7—W1—O4—W3	−89.60 (12)	O4—W2—O8—W3	−0.63 (16)
O5—W1—O4—W3	0.77 (11)	O9—W3—O8—W2	−179.6 (2)
O11—W1—O4—W3	90.10 (12)	O10^i^—W3—O8—W2	−75.4 (2)
O10—W1—O4—W3^i^	0.06 (11)	O13^i^—W3—O8—W2	2.3 (5)
O7—W1—O4—W3^i^	90.40 (12)	O5—W3—O8—W2	76.8 (2)
O5—W1—O4—W3^i^	−179.23 (11)	O4—W3—O8—W2	0.63 (16)
O11—W1—O4—W3^i^	−89.90 (12)	O6—W1—O10—W3^i^	178.9 (2)
O10^i^—W3—O4—W2	90.26 (12)	O7—W1—O10—W3^i^	−76.8 (2)
O13^i^—W3—O4—W2	−179.67 (12)	O5—W1—O10—W3^i^	1.4 (5)
O5—W3—O4—W2	−90.57 (11)	O11—W1—O10—W3^i^	76.5 (2)
O8—W3—O4—W2	−0.46 (12)	O4—W1—O10—W3^i^	−0.09 (16)
O10^i^—W3—O4—W2^i^	−89.74 (12)	O6—W1—O11—W2^i^	178.5 (2)
O13^i^—W3—O4—W2^i^	0.33 (12)	O10—W1—O11—W2^i^	−76.8 (2)
O5—W3—O4—W2^i^	89.43 (11)	O7—W1—O11—W2^i^	0.4 (5)
O8—W3—O4—W2^i^	179.54 (12)	O5—W1—O11—W2^i^	76.4 (2)
O10^i^—W3—O4—W1^i^	0.06 (11)	O4—W1—O11—W2^i^	−0.22 (16)
O13^i^—W3—O4—W1^i^	90.14 (13)	O12—W2—O13—W3^i^	−179.8 (2)
O5—W3—O4—W1^i^	179.23 (11)	O11^i^—W2—O13—W3^i^	−77.1 (2)
O8—W3—O4—W1^i^	−90.66 (13)	O7—W2—O13—W3^i^	75.9 (2)
O10^i^—W3—O4—W1	−179.94 (11)	O8—W2—O13—W3^i^	−0.2 (5)
O13^i^—W3—O4—W1	−89.86 (13)	O4—W2—O13—W3^i^	−0.46 (16)
O5—W3—O4—W1	−0.77 (11)		

Symmetry code: (i) −*x*+1, −*y*, −*z*.
